# Altered hippocampal gene expression and structure in transgenic mice overexpressing neuregulin 1 (*Nrg1*) type I

**DOI:** 10.1038/s41398-018-0288-2

**Published:** 2018-10-22

**Authors:** Inga H. Deakin, Beata R. Godlewska, Mary A. Walker, Guo-Jen Huang, Markus H. Schwab, Klaus-Armin Nave, Amanda J. Law, Paul J. Harrison

**Affiliations:** 1 Department of Psychiatry, University of Oxford, Oxford, UK; 2 Wellcome Trust Centre for Human Genetics, University of Oxford, Oxford, UK; 3 Department of Neurogenetics, Max Planck Institute of Experimental Medicine, Goettingen, Germany; 4 Center of Physiology, Hannover Medical School, Hannover, Germany; 5 Department of Psychiatry, University of Colorado, Denver, USA; 6 Oxford Health NHS Foundation Trust, Oxford, UK

## Abstract

Transgenic mice overexpressing the type I isoform of neuregulin 1 (*Nrg1; NRG1*) have alterations in hippocampal gamma oscillations and an age-emergent deficit in hippocampus-dependent spatial working memory. Here, we examined the molecular and morphological correlates of these findings. Microarrays showed over 100 hippocampal transcripts differentially expressed in *Nrg1*^tg-type I^ mice, with enrichment of genes related to neuromodulation and, in older mice, of genes involved in inflammation and immunity. *Nrg1*^tg-type I^ mice had an enlarged hippocampus with a widened dentate gyrus. The results show that *Nrg1* type I impacts on hippocampal gene expression and structure in a multifaceted and partly age-related way, complementing the evidence implicating *Nrg1* signaling in aspects of hippocampal function. The findings are also relevant to the possible role of *NRG1* signaling in the pathophysiology of schizophrenia or other disorders affecting this brain region.

## Introduction

Neuregulin 1 (*Nrg1*; *NRG1*) is a growth factor, signaling via *Erbb3* and *Erbb4* receptor tyrosine kinases. *Nrg1* plays diverse roles in the development, plasticity, and diseases of the nervous system^1–5^. Its pleiotropy arises, in part, from a family of structurally and functionally distinct isoforms (types I to VI), transcribed from different 5’ exons^6^. In humans, allelic variation can affect NRG1 isoform expression^7–9^, and polymorphisms in *NRG1* may be a risk gene for schizophrenia^10–12^, although this has not been confirmed in genome-wide association studies^13^.

The type I isoform is affected in schizophrenia, with increased expression in hippocampus^7^ and prefrontal cortex^14^ compared with controls, and representing one of the abnormalities of *NRG1-ErbB4* signaling observed in the disorder^15–18^. Reflecting the interest in the functional and pathological roles of *NRG1* type I, a transgenic mouse selectively overexpressing this isoform (*Nrg1*^tg-type I^) was created^19^, and shown to have alterations in myelination^20^ and some aspects of behavior^21^, including an age-emergent deficit in hippocampus-dependent spatial working memory^22^. *Nrg1*^tg-type I^ mice also exhibit a reduced frequency of carbachol-induced hippocampal gamma oscillations^22^. These findings complement a broad body of evidence linking *Nrg1* to hippocampal function and plasticity^23–28^. Differing phenotypic profiles are seen in other genetic mouse models of *Nrg1*, highlighting the existence of isoform-specific properties^29–34^.

In the current study we examined two other aspects of the hippocampus in *Nrg1*^tg-type I^ mice: its transcriptome, and its structure, in comparison with wildtype (wt) littermates. Because of the age-dependent features of the hippocampal phenotype observed previously, the transcriptomic analysis was performed at two ages.

## Materials and methods

All experiments were conducted in accordance with the United Kingdom Animals (Scientific Procedures) Act, 1986, and had local ethical approval.

### Generation and genotyping of *Nrg1*^tg-type I^ mice

The generation and genotyping of the *Nrg1*^tg-type I^ mice has been described^19^. The mice overexpress *Nrg1* type I (β1a-isoform) under a *Thy-1* promoter, with robust overexpression in multiple brain regions, including the hippocampus, with no alteration in *Nrg1* types II or III^20,21^. The experiments reported here were performed in F6-F9 generations of backcross of heterozygous *Nrg1*^tg-type I^ males with wt C57BL/6 J females, comparing *Nrg1*^tg-type I^ mice with their wt littermates.

### Microarrays and quantitative real-time PCR (RT-qPCR)

Two microarray experiments were performed, one in ‘young adult’ mice (2.5–4 months), the other in ‘old’ mice (14–15 months). Each comprised 24 animals, 6 of each genotype and sex.

#### RNA extraction and preparation for microarrays

Brains were frozen in isopentane cooled on dry ice. The left hemisphere was placed into RNAlater®-ICE Frozen Tissue Transition Solution (Ambion) at − 20 °C for 18 h, after which the hippocampus was dissected and homogenized in Qiazol in a TissueLyser (Qiagen). Total RNA was extracted and purified with RNeasy lipid tissue Mini kits (Qiagen) according to manufacturer’s protocol. In total, 300 ng RNA was used for amplification and labeling with Illumina® TotalPrep™ RNA Amplification Kit (Ambion). Complementary RNA (cRNA) quality was determined with an Agilent 2100 Bioanalyser (Agilent Technologies, Palo Alto, CA).

A total of 1.5 µg cRNA from each brain was hybridized to an Illumina Mouse WG-6 v1.1 (young mice) or v2 (old mice) chip according to manufacturers’ protocol and scanned with a BeadStation 500 machine.

#### Microarray analysis

Standard quality control measures were performed with the BeadStudio program (Illumina, CA), including subtracting the background from each array from the raw signal intensity of each probe type. The raw signal intensity data underwent variance stabilizing normalization, which is a generalized log_2_ transformation of the signal. A quantile standardization procedure was used to centralize the mean signal in the distribution (i.e., of all the probes) and to equalize the variance between mice. Further quality control was performed such as hierarchical clustering and box plots of normalized intensity (robust multichip average; RMA) values for each chip.

A linear model with an empirical Bayes *t*-statistic was fitted to the data to generate lists of significant effects of genotype, using the Limma program^35^. We corrected for multiple testing with the Benjamini–Hochberg false discovery rate to give an adjusted *p* value^36^. The criteria for differential expression were an adjusted *p* value < 0.05 and an absolute (unlogged RMA) fold change in expression (FC) > 1.5.

Differentially expressed probe lists were condensed into lists of differentially expressed transcripts, including known splice variants, identified with PubMed basic local alignment search tool (BLAST). Probes with no accession number were identified by nucleotide sequence with PubMed Nucleotide BLAST. Results from each array were compared with obtain lists of genes that were differentially expressed in *NRG1*^tg-type I^ mice at both ages, or only at one of the two ages. These three lists of genes were used for further investigation with Ingenuity Pathways Analysis (IPA; Ingenuity Systems), which generated networks of up to 35 genes based on their known functional links^37^.

#### RT-qPCR

Hippocampal RNA was treated with 1 μl (1 unit) RQ1 RNase-free DNAse (Promega) and 0.6 μl (24 units) RNasin ribonuclease inhibitor (Promega) at 37 °C for 30 min and then heated to 72 °C for 10 min. DNAse-treated RNA was reverse-transcribed using 1 μl (200 units) MMLV reverse transcriptase, Promega), 1xMMLV buffer, 0.8 μl of 10 mM dNTPs, 0.6 μl (24 units) RNasin, 0.6 μl of 10 mM oligoDTs. The reaction mix was incubated at 42 °C for 1 h and then heated to 72 °C for 10 min. The reverse-transcribed RNA (complementary DNA; cDNA) was diluted with nuclease-free water and stored at − 80 °C.

TaqMan assays were ordered from Applied Biosystems (*Npy*, Mm00445771_m1; *Gfap*, Mm01253034_m1; *Inhba*, Mm00434339_m1; *Cntfr*, Mm00516697_m1; *C1qa*, Mm00432142_m1; Rbbp4, Mm00771401_g1). *Bdnf* was detected using pan-BDNF primers and a TaqMan probe (Forward, 5′-GGGTCACAG CGGCAGATAAA-3′, Reverse 5′-GCCTTTGGATACCGGGACTT-3′; Probe, TCTGGCGGGACGGTCACAGTCCTA)^38^. *Bdnf* v1-specific probes^39^ were: Forward, 5′-CACATTACCTTCCTGCATCTGTTG-3′, reverse 5′-ACCATAGTAAGGAAAAGGATGGTCAT-3′, probe AAGCCACAATGTTCCACCAG. The PCR reaction mix included 15 ng cDNA, 1xTaqMan Universal PCR Master Mix (Applied Biosystems) and the assay in a final volume (with nuclease-free water) of 15 μl in 384-well plates. *Nrg1* mRNA was quantified using a SyBr green assay, with the primers designed so that the PCR product would span the exon boundary between the type I-specific exon and the immunoglobulin-like domain, and checked in the BLAST database for binding specificity (Forward, 5′-AAGGGGAAGGGCAAGAAGAA-3′, Reverse 5′-TCTTTCAATCTGGGAGGCAAT-3′; Eurogentech). The reaction mix for *Nrg1* type I was 1xSyBr Green Mix, 15 ng cDNA, 200 nM of each primer and nuclease-free water up to a final volume of 15 μl.

Standard curves of pooled cDNA from all samples were set up in triplicate with the starting amount of cDNA ranging from 100 ng to 0.54 pg (*Nrg1*), 5 pg (*Npy*), 0.01 ng (*Gfap*), or 0.39 ng (*Inhba, Cntfr, C1qa, Rbbp4*). The *R*^2^ of all standard curves was > 0.99. All experimental samples fell within the standard curve. Samples were run in triplicate on the same plate as the standard curve. No-template controls and RT-negative controls were also run in triplicate to test for any contamination of the reaction mix or cDNA, respectively. Cycling conditions for all qRT-PCR reactions were 50 °C for 2 min, 95 °C for 10 min and then 40 repeats of 95 °C for 15 seconds to denature and 60 °C for 1 min.

### Morphology and histology

Snap-frozen brains from 10-month-old wt and *Nrg1*^tg-type I^ mice were coronally cryosectioned at 20 μm thickness. Hippocampal area was measured by point counting on multiple cresyl violet-stained sections throughout the dorsoventral extent, and hippocampal volume estimated using Cavalieri’s theorem as described^40^. Whole brain volume was estimated in the same way. We also measured the cross-sectional area of subfields (dentate gyrus, CA3, CA1) by manual tracing using a Nikon Eclipse 3600 microscope coupled to an MCID Elite image analysis system (Interfocus, Haverhill, UK). Other sections were immunostained for parvalbumin (PV27, 1:100; Swant, Switzerland), detected with diaminobenzidine, by standard methods. All measurements were made blind to genotype.

## Results

### The hippocampal transcriptome of *Nrg1*^tg-type I^ mice: age-related effects on genes involved in myelination, neurotransmission, and immunity

**Fig. 1:**
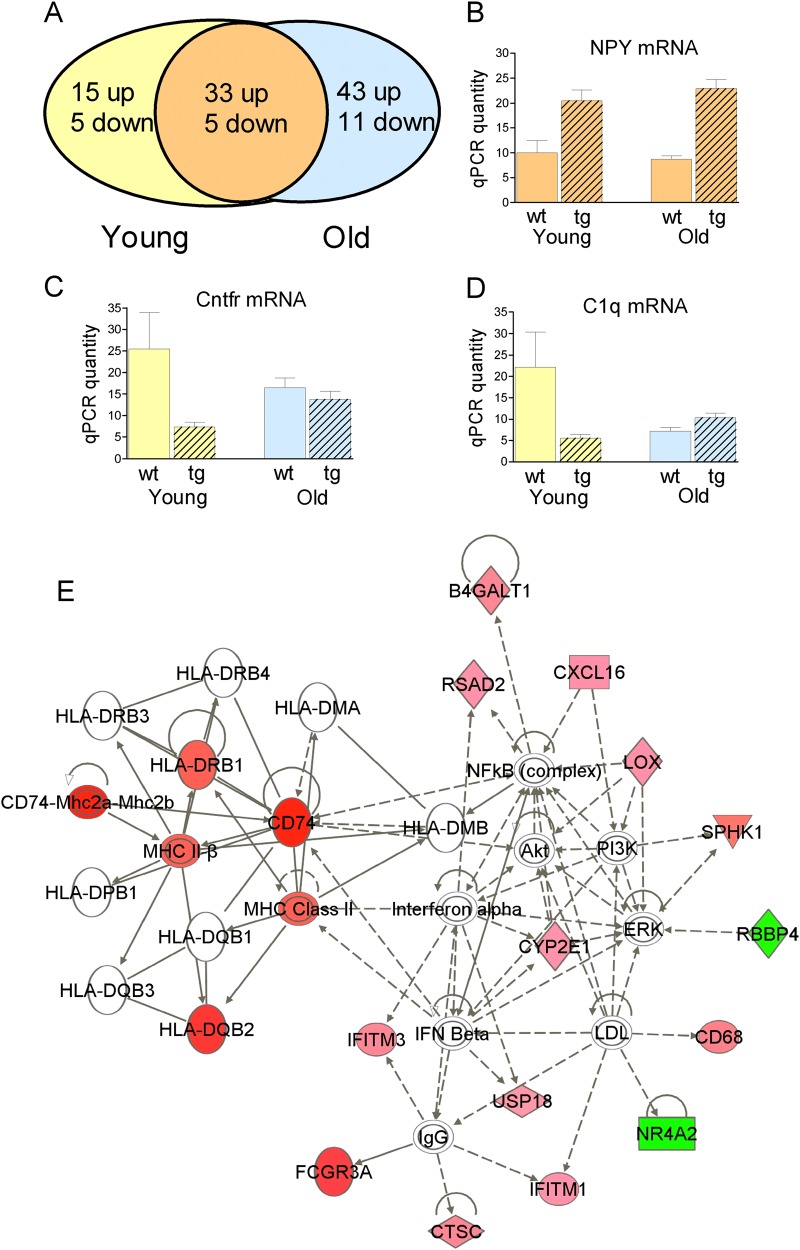
Hippocampal gene expression in *Nrg1*^tg-type I^ mice. **a** Genes meeting the criteria described in text for differential expression in young (yellow) or old (blue) adult *Nrg1*^tg-type I^ mice, or *Nrg1*^tg-type I^ mice of both ages (orange). **b**–**d** examples of RT-qPCR validation of transcripts from each group. **b** *Npy* mRNA, increased in *Nrg1*^tg-type I^ mice of both ages; **c** *Cntfr* mRNA, decreased in young but not old adult *Nrg1*^tg-type I^ mice; **d** *C1q* mRNA, increased in old but not young adult *Nrg1*^tg-type I^ mice. Statistics for the data shown in panels **b**–**d** are given in Table 1. **e** An IPA network of transcripts differentially expressed in old but not young adult *Nrg1*^tg-type I^ mice. The network comprises nodes (genes) and their biological relationships shown by interconnecting lines. Red nodes are transcripts with increased expression, and the green nodes are transcripts with lower expression, in the old *Nrg1*^tg-type I^ mice, compared with their age-matched wt controls. Increasing color intensity indicates a greater fold change. White nodes show genes that are functionally related to the other differentially expressed genes in the network and added by IPA. Solid lines between nodes indicate a direct interaction between them and dashed lines indicate indirect relationships. A continuous line denotes “binding only”; pointed line, “acts upon” and blunt ended line, “inhibits”. For gene symbols and names, see Supplementary Table 4. For additional IPA networks identified in one or both age groups of *Nrg1*^tg-type I^ mice, see Supplementary Tables 1–3 and Supplementary Figures 1–4

*Nrg1* impacts upon the expression of many individual genes^1,3^. Here we used microarrays as an unbiased method to identify transcripts and networks altered in the hippocampus of *Nrg1*^tg-type I^ mice. Adopting a stringent statistical approach, and with a 1.5-fold change threshold, we identified over 100 differentially expressed genes, of which ~ 80% were increased in *Nrg1*^tg-type I^ mice compared with wt (Fig. 1a, and Supplementary Tables 1–3). Thirty-eight transcripts were altered in the same direction at both ages (Supplementary Table 1), 20 were differentially expressed only in the young adult (2.5–4 month) *Nrg1*^tg-type I^ mice (Supplementary Table 2) and 54 only in old (14–15 month) *Nrg1*^tg-type I^ mice (Supplementary Table 3). Eight transcripts were selected for qPCR validation, based on the microarray results as well as what was known about their functions and interactions with Nrg1 (Fig. 1b–d; and Table 1).

**Table 1:** Quantitative RT-PCR validation of differentially expressed genes in *Nrg1*^tg-type I^ mice

		Young adult (2.5–4 months)				Old (4–15 months)			
		Microarray		qRT-PCR		Microarray		qRT-PCR	
Accession	Gene	FC	*p*	FC	*p*	FC	*p*	FC	*p*
	NRG1 type I	8.60	2.62E-19	801	0.001	NA	NA	466	0.001
NM_023456.2	NPY	2.22	1.01E-05	2.0	0.005	2.46	9.93E-06	2.6	0.001
NM_010277	GFAP	1.59	1.69E-03	NC	NC	1.62	1.42E-02	1.5	0.036
NM_007540.3	BDNF v1	1.65	4.12E-04	3.5	0.001	2.76	8.00E-07	NC	NC
NM_008380.1	Inhba	2.28	4.83E-06	NC	NC	NC	NC	2.7	0.001
NM_016673.1	Cntfr	−1.86	5.66E-03	−3.4	0.068	NC	NC	NC	NC
NM_007572	C1qa	NC	NC	NC	NC	1.60	6.14E-04	1.4	0.032
NM_009030	Rbbp4	NC	NC	NC	NC	−1.89	6.14E-04	NC	NC

NA: probe not present on array. NC: no significant change. The statistical approach to the microarray data is described in text; *p* values for qRT-PCR are from unpaired t tests (two-tailed) comparing transgenic and wt mice of each age group

Genes upregulated at both ages in the *NRG1*^tg-type I^ mice included neuropeptide Y (*Npy*), brain-derived neurotrophic factor (*Bdnf*), and glial fibrillary acidic protein (*Gfap*). Consistent with the *Npy* mRNA data, Npy-immunoreactive hippocampal interneurons were markedly more prominent in the *Nrg1*^tg-type I^ mice (not shown). The *Bdnf* mRNA increase affected the V1 isoform selectively. IPA generated two networks with scores corresponding to significance values of *p* = 10^−31^ and *p* = 10^−25^ (Supplementary Figs. 1 and 2). Network 1 included, as well as *Npy* and *Bdnf*, several transcripts involved in neurotransmission and implicated in schizophrenia, such as dopamine D1 and D4 receptors (*Drd1* and *Drd4*).

Of the genes differentially expressed in young but not old *Nrg1*^tg-type I^ mice, three were involved in myelination (myelin basic protein (Mbp), myelin oligodendrocyte glycoprotein (*Mog)*, and myelin-associated oligodendrocytic basic protein). IPA generated a network that included *Mog* and *Mbp* (*p* = 10^−35^; Supplementary Fig. 3).

Genes overexpressed in old but not young *Nrg1*^tg-type I^ mice (Supplementary Table 3) included many with immune and inflammatory functions, and IPA generated two highly significant networks (*p* = 10^−37^ and *p* = 10^−34^; one is shown in Fig. 1e, the other in Supplementary Fig. 4). In particular, the network shown in Fig. 1e includes many upregulated HLA and major histocompatibility complex (MHC) genes (Supplementary Table 4).

### *Nrg1*^tg-type I^ mice have altered hippocampal morphology

**Fig. 2:**
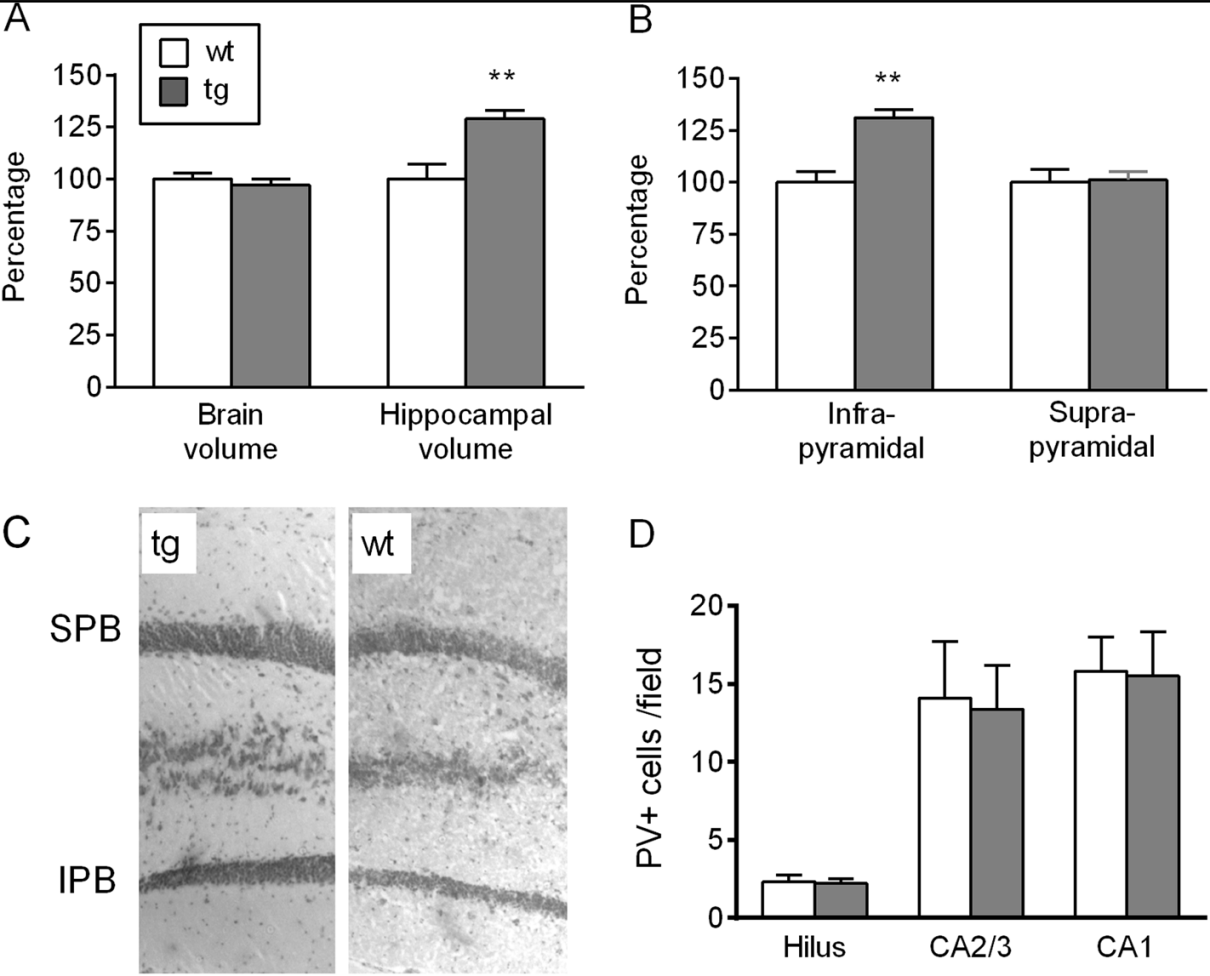
Hippocampal morphology in *Nrg1*^tg-type I^ mice. **a** Hippocampal volume is increased in *Nrg1*^tg-type I^ mice (*n* = 10) compared with wt (*n* = 9; two-tailed unpaired *t* test, *t* = 3.249, df = 17, *p* = 0.006), but whole brain volume is unchanged. **b** The width of the dentate gyrus granule cell layer is increased in *Nrg1*^tg-type I^ mice in the infra-pyramidal blade (*n* = 6 in each group; two-tailed unpaired *t* test, *t* = 4.126, df = 10, *p* = 0.002) but not in the supra-pyramidal blade. **c** Illustration of the data in **b**, showing the wider infra-pyramidal blade (IPB) in a *Nrg1*^tg-type I^ mouse compared with a wt mouse. The supra-pyramidal blade (SPB) is also shown, with CA3 in between. **d** The density of parvalbumin (PV)-immunoreactive cells did not differ in *Nrg1*^tg-type I^ mice (*n* = 10) compared with wt (*n* = 12). Bars in **a**, **b**, and **d** show mean and standard deviation. All data in this Figure come from 10-month-old mice

As shown in Fig. 2a, the hippocampus was enlarged (by ~ 25%) in *Nrg1*^tg-type I^ mice, with brain volume unchanged. In terms of individual subfields, the only difference observed in *Nrg1*^tg-type I^ mice was that the dentate gyrus granule cell layer was wider than in wt mice; this was primarily in the infra-pyramidal (external) blade rather than the supra-pyramidal (internal) blade (Fig. 2b, c). The density of PV + interneurons was unchanged in each subfield measured (Fig. 2d).

## Discussion

Mice selectively overexpressing the type I isoform of *Nrg1* show differences in hippocampal function and age-emergent deficits in hippocampus-dependent behavior^22^. Here, we report that these changes are accompanied by an altered profile of gene expression which differs between young adult (2.5–4 month) and old (14–15 month) *Nrg1*^tg-type I^ mice, and by an increase in hippocampal volume.

### The hippocampal molecular and morphological profile of *Nrg1*^tg-type I^ mice

*Nrg1*^tg-type I^ mice exhibited differential expression of a number of genes (Fig. 1; Table 1; Supplementary Figures 1–4; Supplementary Tables 1–3). Of the genes overexpressed in *Nrg1*^tg-type I^ mice at both ages, several are noteworthy. In particular, five transcripts (*Npy, Gfap, Bdnf, Drd1*, and *Drd4*) were part of the most significant gene network and all had been linked previously to *Nrg1*. The fact they were upregulated in both age cohorts, which were studied separately and with different versions of the microarray chip, strengthens the robustness of the findings.

*Npy* is expressed by a subpopulation of hippocampal interneurons, bistratified and ivy cells, which impose a strong inhibitory influence on pyramidal cell dendrites^41,42^. Its marked upregulation in the *Nrg1*^tg-type I^ mice (Fig. 1b) is of interest for several reasons. First, it provides another hint that interneurons are affected and may contribute to the oscillatory and circuitry alterations that underlie the phenotype of the mice^41–44^. Second, *Npy* is anti-epileptic^45–47^, and enhanced *Npy* expression may help prevent the epileptiform predisposition of *Nrg1*^tg-type I^ mice^22^ progressing to overt seizure activity. Third, the *Npy* mRNA elevation may be related to the morphological finding of a widened dentate gyrus. Preliminary data show an increase of hilar cells immunoreactive for doublecortin, a marker of newly formed neurons, in the *Nrg1*^tg-type I^ mice (I.H.D. and P.J.H., unpublished observations), suggesting that the enlarged dentate gyrus might reflect increased adult neurogenesis—a process stimulated by *Npy*^48–50^, and influenced by *Nrg1*^51^. Whether the persisting upregulation of *Gfap* mRNA in *Nrg1*^tg-type I^ mice (Table 1) is also indicative of enhanced cell proliferation—as many neural precursors express *Gfap*^52^—remains to be seen; it might also be a remnant of the developmental role of *Nrg1-ErbB* signaling in neuron–astrocyte differentiation^53,54^. *Bdnf* is a regulator of hippocampal plasticity and function^55^, and its elevated expression may have many manifestations in the *Nrg1*^tg-type I^ mice, including a contribution to their spatial working memory deficit^22,56^. Finally, the upregulation of *Drd1* and *Drd4* mRNAs complements evidence that hippocampal *Drd4* mediates *Nrg1*-induced reversal of LTP^24^, and that Nrg1 application produces acute^57^ and sustained^58^ increases in dopamine release and dopamine neuron firing^59^. Another *Nrg* genetic mouse model also shows dopamine receptor alterations^60^.

The gene expression differences that occurred in the old but not young *Nrg1*^tg-type I^ were striking, comprising many immune and inflammatory genes, such as HLA-DR, MHC class II CD74, and complement *C1q*. Similar changes have been reported in old vs. young wt rodents and in this respect there may be an ‘accelerated aging’ phenotype in *Nrg1*^tg-type I^ mice^61–64^. *C1q*, like other complement factors, is also involved in neuronal and synaptic function^65–67^ and dysfunction^68–70^ and brain aging^71^. As such, the altered expression of the genes in the older *Nrg1*^tg-type I^ mice may represent molecular correlates of, and might contribute to, their age-emergent memory impairment.

Fewer genes were differentially expressed in the young but not old *Nrg1*^tg-type I^ mice, and they were primarily myelin-related. This is not unexpected, in that *Nrg1* is a critical player in myelination^72,73^. Although the type I isoform has hitherto been implicated primarily in peripheral myelination^19,74,75^, these mice do show hypermyelination of small diameter axons in the central nervous system^20^. The upregulation of myelin-related transcripts in the *Nrg1*^tg-type I^ mice may be a molecular indication that this process is also occurring in the hippocampus; any resulting hypermyelination may in turn contribute to the hippocampal volume increase. However, this remains speculative; indeed, more generally, the processes that link the morphological and molecular alterations reported here remain unknown.

The altered transcriptomic profile of the *Nrg1*^tg-type I^ mice highlights an issue that pertains broadly to genetically modified animals: their phenotypes need not arise solely from the targeted gene(s) but also from the cascade of molecular changes which the manipulation induces. Moreover, these effects are not static but vary with age, and illustrate the value of going beyond the 3–6 month time-point at which characterization is often completed, even though capturing the temporal dynamic and longitudinal profile is demanding of resources and time.

### Implications for NRG1 in schizophrenia

The evidence mentioned earlier showing *Nrg1*-dopamine interactions, and the increased D1 and D4 receptor expression seen here is notable, given that dopaminergic abnormalities are a final common pathway in schizophrenia pathophysiology^76^, and alterations in both receptors have been reported in the disorder^77,78^. The MHC complex is strongly implicated in schizophrenia^79^, in part through the complement *C4* gene^80^, and several^81–84^ though not all^85^ studies report elevated expression of many immune and inflammatory genes, including *C1q*, especially in older, chronically ill patients^86^, reminiscent of the gene expression changes being restricted to the old *Nrg1*^tg-type I^ mice. There is also some evidence for direct links between *NRG1*, immune function, and schizophrenia^87–89^.

However, there is less congruence when other findings are considered. Thus, in schizophrenia, in contrast to findings in the *Nrg1*^tg-type I^ mice, hippocampal myelin-related transcripts are decreased^90^, hippocampal volume is unchanged or reduced^91^, and there is a lower density of hippocampal PV + neurons^92^. Thus, although the phenotype of *Nrg1*^tg-type I^ mice may be seen as overlapping to an extent with that of schizophrenia, the differences are at least as striking as the similarities. One specific factor to consider is that the magnitude of overexpression in the *Nrg1*^tg-type I^ mouse is far greater than the increased *NRG1* type I expression reported in schizophrenia. More generally, these considerations draw attention to the need for cautious interpretation and extrapolation from any genetic mouse model to the human syndrome. Nevertheless, the results extend the evidence that *Nrg1* type I is functional in the hippocampus, and hence may play a role in any disease in which its expression in this brain region is altered.

## Electronic supplementary material

Supplementary Materials
