# Supplementary material for: Altered hippocampal gene expression and structure in transgenic mice overexpressing neuregulin 1 (Nrg1) type I
Source: Transl Psychiatry. 2018 Oct 22;8:229. doi: 10.1038/s41398-018-0288-2 (PMC6197224; doi:10.1038/s41398-018-0288-2)
Supplement: Supplementary file 1 — Supplementary Materials [file 41398_2018_288_MOESM1_ESM.doc]

**Alterations in Hippocampal Gene Expression and Structure in Transgenic Mice Over-expressing Neuregulin 1 (NRG1) type I**

by *Deakin et al.*

**Supplementary Material**

*Contents*

1. Supplementary Tables
2. Supplementary Figures

**1. Supplementary Tables**

**Supplementary Table 1.** Genes differentially expressed (adjusted p<0.05 and FC >1.5) in both young and old NRG1^tg-type I^ mice. Genes in grey are down-regulated in NRG1^tg-type I^ mice, all others are up-regulated in NRG1^tg-type I^ mice compared to their wildtype littermates. FC, fold change.

|  |  |  |  | YOUNG | | OLD | |
| --- | --- | --- | --- | --- | --- | --- | --- |
|  | Definition | Symbol | Accession | FC | adj-p | FC | adj-p |
| 1 | Thymus cell antigen 1, theta | Thy1 | NM_009382.2 | 2.18 | 3.74E-11 | 2.13 | 1.05E-07 |
| 2 | Fibulin 2, transcript variant 1 | Fbln2 | NM_007992.1 | 2.36 | 2.41E-09 | 3.58 | 1.72E-06 |
| 3 | Activin A receptor, type IC | Acvr1c | NM_001033369.2 | 4.34 | 8.50E-08 | 3.53 | 3.33E-07 |
| 4 | Preproenkephalin 1 | Penk1 | NM_001002927.2 | 2.46 | 8.50E-08 | 2.22 | 9.90E-05 |
| 5 | Agmatine ureohydrolase (agmatinase) | Agmat | NM_001081408.1 | 2.45 | 2.79E-06 | 4.99 | 6.02E-11 |
| 6 | Serine (or cysteine) peptidase inhibitor, clade A, member 3N | Serpina3n | NM_009252.1 | 2.01 | 4.62E-06 | 2.25 | 1.74E-06 |
| 7 | Basic leucine zipper transcription factor, ATF-like 3 | Batf3 | NM_030060.1 | 2.36 | 4.62E-06 | 4.11 | 1.49E-06 |
| 8 | Neuropeptide Y | Npy | NM_023456.2 | 2.22 | 1.01E-05 | 2.46 | 9.93E-06 |
| 9 | Dopamine receptor D1A | Drd1a | NM_010076 | 2.44 | 2.40E-05 | 2.13 | 1.19E-05 |
| 10 | Transmembrane channel-like gene family 6, transcript variant 1 | Tmc6 | NM_145439.1 | 1.75 | 2.40E-05 | 2.50 | 2.63E-05 |
| 11 | Glycoprotein (transmembrane) nmb | Gpnmb | NM_053110.2 | 2.45 | 2.40E-05 | 4.21 | 2.51E-05 |
| 12 | Neuronal pentraxin 2 | Nptx2 | NM_016789.2 | 2.20 | 2.56E-05 | 2.46 | 2.27E-03 |
| 13 | Reprimo, TP53 dependent G2 arrest mediator candidate | Reprimo | NM_023396.3 | 2.51 | 3.92E-05 | 1.69 | 1.65E-02 |
| 14 | Insulin-like growth factor binding protein 6 | Igfbp6 | NM_008344.1 | 2.09 | 5.10E-05 | 2.73 | 7.94E-05 |
| 15 | Pleckstrin homology domain-containing, family A (phosphoinositide binding specific) member 2 | Plekha2 | NM_031257.2 | 2.47 | 5.29E-05 | 2.32 | 2.14E-04 |
| 16 | Kv channel interacting protein 3, calsenilin | Kcnip3 | NM_019789.2 | 1.57 | 5.72E-05 | 1.65 | 6.14E-04 |
| 17 | K+ voltage-gated channel, subfamily S, 2 | Kcns2 | NM_181317.3 | 1.69 | 1.25E-04 | 1.66 | 1.86E-04 |
| 18 | Periplakin | Ppl | NM_008909.2 | 1.64 | 1.70E-04 | 1.68 | 2.78E-04 |
| 19 | Enoyl Coenzyme A hydratase domain containing 2 | Echdc2 | NM_026728.1 | -1.62 | 1.93E-04 | -2.67 | 3.40E-06 |
| 20 | B-cell linker | Blnk | NM_008528.3 | 1.79 | 1.95E-04 | 4.26 | 1.06E-05 |
| 21 | HtrA serine peptidase 4 | Htra4 | XM_284398.1 (BLASTs NM_001081187) | 2.05 | 2.46E-04 | 2.17 | 5.93E-04 |
| 22 | Solute carrier family 23 (nucleobase transporters),member 3 | Slc23a3 | NM_194333.2 | 2.14 | 3.52E-04 | 4.25 | 3.33E-07 |
| 23 | Lipoma HMGIC fusion partner | Lhfp | NM_175386.3 | 1.52 | 3.57E-04 | 1.72 | 8.61E-03 |
| 24 | Zinc finger, CCHC domain containing 5 | Zcchc5 | NM_199468.1 | -1.78 | 3.67E-04 | -2.23 | 9.75E-04 |
| 25 | Brain derived neurotrophic factor, transcript variant 1 | Bdnf | NM_007540.3 | 1.65 | 4.12E-04 | 2.76 | 8.00E-07 |
| 26 | Calsenilin, presenilin binding protein, EF hand transcription factor | Csen | NM_019789.2 | 1.65 | 6.14E-04 | 1.57 | 5.72E-05 |
| 27 | Downstream of Stk11 | Dos | NM_015761.1 | -1.51 | 8.57E-04 | -1.52 | 1.76E-02 |
| 28 | Myosin, light polypeptide kinase | Mylk | NM_139300 | 1.50 | 1.35E-03 | 1.82 | 1.24E-03 |
| 29 | Glial fibrillary acidic protein, transcript variant 2 | Gfap | NM_010277 | 1.59 | 1.69E-03 | 1.62 | 1.42E-02 |
| 30 | BLASTS genomic contigs | A930033C01Rik | XM_132396.4 | 1.58 | 2.14E-03 | 1.76 | 1.31E-03 |
| 31 | Kallikrein related-peptidase 8 (Klk8) | Prss19 | NM_008940.1 | 1.77 | 2.33E-03 | 1.64 | 4.70E-02 |
| 32 | Olfactomedin 4 | Olfm4 | NM_001030294.1 | 1.77 | 3.26E-03 | 1.78 | 3.40E-05 |
| 33 | Dopamine receptor 4 | Drd4 | NM_007878 | 1.61 | 3.49E-03 | 2.90 | 2.99E-05 |
| 34 | Otospiralin | Otos | NM_153114.1 | 1.66 | 3.67E-03 | 2.99 | 4.73E-04 |
| 35 | PDZ and LIM domain 1 (elfin) | Pdlim1 | NM_016861 | 1.53 | 1.65E-02 | 1.83 | 7.72E-04 |
| 36 | Arylsulfatase J | Arsj | NM_173451.1 | 1.67 | 2.66E-02 | 1.98 | 2.22E-03 |
| 37 | Ryanodine receptor 1 | Ryr1 | NM_009109 | -1.55 | 3.31E-02 | -1.67 | 1.39E-02 |
| 38 | BLASTS genomic sequences- only 30% CXXC finger 6, transcript variant 2 | Cxxxc6 | 4933439C10Rik | -1.51 | 1.59E-04 | -1.93 | 3.74E-05 |

**Supplementary Table 2**. Genes differentially expressed in young (adj-p<0.05 and FC>1.5) but not old (unadjusted p>0.1) NRG1^tg-type I^ mice. Genes in grey are down-regulated in NRG1^tg-type I^ mice, all others are up-regulated in NRG1^tg-type I^ mice compared to their wildtype littermates. FC, fold change.

|  |  |  |  | *YOUNG* | |
| --- | --- | --- | --- | --- | --- |
|  | Definition | Name | Accession | FC | adj-p |
| 1 | Inhibin beta-A | Inhba | NM_008380.1 | 2.28 | 4.83E-06 |
| 2 | Thrombospondin, type I, domain containing 7B | Thsd7b | NM_172485.2 | 1.56 | 1.59E-04 |
| 3 | RIKEN cDNA E130012A19 gene | E130012A19Rik | NM_175332.3 | -1.66 | 3.52E-04 |
| 4 | Lysophosphatidic acid receptor 1, transcript variant 1 | Lpar1 | NM_010336.1 | 1.56 | 8.42E-04 |
| 5 | BLASTS- similar to 4933409K07Rik protein - which is XR_030661.1, hypothetical protein LOC108816 | LOC331139 | XM_284587.2 | -1.82 | 9.59E-04 |
| 6 | Transmembrane protein with EGF-like and two follistatin-like domains 2 | Tmeff2 | NM_019790.2 | 1.57 | 1.62E-03 |
| 7 | Transcription elongation regulator 1-like | Tcerg1l | NM_183289 | -1.61 | 2.74E-03 |
| 8 | Aldehyde dehydrogenase family 1, subfamily A1 | Aldh1a1 | NM_013467 | 1.55 | 4.72E-03 |
| 9 | Ciliary neurotrophic factor receptor | Cntfr | NM_016673.1 | -1.86 | 5.66E-03 |
| 10 | Expressed sequence AI427122 (AI427122) | AI427122 | XM_110660.4 | 1.56 | 6.05E-03 |
| 11 | Myelin oligodendrocyte glycoprotein | Mog | NM_010814.1 | 1.57 | 7.56E-03 |
| 12 | Suppression of tumorigenicity 18 | St18 | NM_173868.1 | 1.62 | 1.19E-02 |
| 13 | Myelin-associated oligodendrocytic basic protein, transcript variant 3 | Mobp | NM_001039365.1 | 1.56 | 1.32E-02 |
| 14 | Tachykinin 1 | Tac1 | NM_009311.1 | -1.67 | 1.38E-02 |
| 15 | Galanin | Gal | NM_010253 | 1.62 | 2.24E-02 |
| 16 | Myelin basic protein, transcript variant 8 | Mbp | NM_001025245.1 | 1.50 | 2.75E-02 |
| 17 | Poliovirus receptor-related 3, transcript variant alpha | Pvrl3 | NM_021495 | 1.78 | 3.21E-02 |
| 18 | Anillin, actin binding protein (scraps homolog, Drosophila) | Anln | NM_028390.1 | 1.53 | 3.75E-02 |
| 19 | Tetraspanin 2 | Tspan2 | NM_027533 | 1.59 | 3.85E-02 |
| 20 | Fatty acid 2-hydroxylase | Fa2h | NM_178086.2 | 1.55 | 4.10E-02 |

**Supplementary Table 3**. Genes differentially expressed in old (adj-p<0.05 and FC>1.5) but not young (unadjusted p>0.1) NRG1^tg-type I^ mice. Genes in grey are down-regulated in NRG1^tg-type I^ mice, all others are up-regulated in NRG1^tg-type I^ mice compared to their wildtype littermates. FC, fold change.

|  |  |  |  | OLD | |
| --- | --- | --- | --- | --- | --- |
|  | Definition | Symbol | Accession | FC | adj-p |
| 1 | Fc receptor-like 3 | Fcrl3 | NM_144559.1 | 2.73 | 8.00E-07 |
| 2 | Similar to embryonic blastocoelar extracellular matrix protein precursor | LOC333315 | XM_286119.2 | -2.60 | 5.00E-05 |
| 3 | Chemokine (C-X-C motif) ligand 16 | Cxcl16 | NM_023158.3 | 1.68 | 5.83E-05 |
| 4 | Pregnancy-associated plasma protein A | Pappa | NM_021362 | 2.10 | 9.31E-05 |
| 5 | Response to metastatic cancers 1 | Rmcs1 | NM_207105.1 | 2.84 | 3.74E-04 |
| 6 | RIKEN cDNA 1300007C21 gene (1300007C21Rik), BLASTs hypothetical proteins LOC100041156 AND LOC100041932 | 1300007C21Rik | NM_175119.1 | -1.92 | 4.37E-04 |
| 7 | Hypothetical protein, OK in DAVID | E030003B04Rik | AK086820 | -1.68 | 5.93E-04 |
| 8 | Interleukin 28 receptor alpha | Il28ra | NM_174851.2 | 1.57 | 5.94E-04 |
| 9 | Retinoblastoma binding protein 4 | Rbbp4 | NM_009030 | -1.89 | 6.14E-04 |
| 10 | Complement component 1, q subcomponent, alpha polypeptide | C1qa | NM_007572 | 1.60 | 6.14E-04 |
| 11 | Hypothetical Microbodies C-terminal targeting signal containing protein, BLASTs genomic | 9430091F09Rik | AK035122 | -1.75 | 7.72E-04 |
| 12 | Bst2 bone marrow stromal cell antigen 2 | Bst2 | NM_198095.1 | 2.18 | 8.20E-04 |
| 13 | CD68 antigen | Cd68 | NM_009853.1 | 1.87 | 1.21E-03 |
| 14 | Protocadherin 21 | Pcdh21 | NM_130878.2 | -1.87 | 1.28E-03 |
| 15 | ATPase, Ca++ transporting, cardiac muscle, fast twitch 1 | Atp2a1 | NM_007504.2 | 1.98 | 1.57E-03 |
| 16 | Interferon induced transmembrane protein 3 | Ifitm3 | NM_025378.1 | 1.80 | 1.74E-03 |
| 17 | Ia-associated invariant chain | Ii | NM_010545.2 | 3.05 | 2.22E-03 |
| 18 | Unc5d unc-5 homologueD – D930029E11Rik - NETRIN receptor | unc5d | NM_153135 | 1.69 | 2.22E-03 |
| 19 | Crystallin, beta A2 | Cryba2 | NM_021541.1 | 1.86 | 3.45E-03 |
| 20 | Histocompatibility 2, class II antigen E beta | H2-Eb1 | NM_010382.1 | 2.29 | 3.50E-03 |
| 21 | RIKEN cDNA E130203B14 gene | E130203B14Rik | NM_178791.2 | 1.69 | 4.17E-03 |
| 22 | ATP binding domain 1 family, member B | Atpbd1b | NM_133884.1 | -1.57 | 4.55E-03 |
| 23 | Olfactory receptor 1348 | Olfr1348 | NM_146913 | 1.94 | 5.39E-03 |
| 24 | Interferon induced transmembrane protein 1 | Ifitm1 | NM_026820 | 1.65 | 5.61E-03 |
| 25 | Gliomedin | Gldn | NM_177350.2 | 1.76 | 6.78E-03 |
| 26 | Myosin IF | Myo1f | NM_053214.1 | 1.50 | 7.80E-03 |
| 27 | Junctional sarcoplasmic reticulum protein 1 - 2300003C06Rik | 2300003C06Rik -Jsrp1 | NM_028001.1 | 1.56 | 8.41E-03 |
| 28 | PREDICTED: meiosis defective 1, transcript variant 4 | Mei1 | XM_901649 | 1.58 | 1.00E-02 |
| 29 | Epithelial membrane protein 3 | Emp3 | NM_010129 | 1.76 | 1.17E-02 |
| 30 | No information, OK in DAVID | 9030224M15Rik | NM_177793.2 | 2.09 | 1.31E-02 |
| 31 | Claudin | Cldn22 | NM_029383.1 | -1.58 | 1.31E-02 |
| 32 | Lysyl oxidase | Lox | NM_010728.1 | 1.67 | 1.38E-02 |
| 33 | Annexin A2 | Anxa2 | NM_007585.2 | 1.73 | 1.44E-02 |
| 34 | Nuclear receptor subfamily 4, group A, member 2, (Nurr1) | Nr4a2 | NM_013613 | -2.00 | 1.58E-02 |
| 35 | no information, RIKEN gene cDNA 9030224M15Rik gene, OK in DAVID | A130026C10Rik | AK037550 | 1.58 | 1.58E-02 |
| 36 | CBFA2T1 identified gene homolog (human) | Cbfa2t1h | NM_009822.1 | -1.62 | 1.65E-02 |
| 37 | Sphingosine kinase 1 | Sphk1 | NM_011451.1 | 2.02 | 1.79E-02 |
| 38 | Cytochrome P450, family 2, subfamily e, polypeptide 1 | Cyp2e1 | NM_021282.1 | 1.63 | 1.94E-02 |
| 39 | Leucine rich repeat containing 55 - A330097E02Rik | Lrrc55 | NM_001033346.2 | 1.57 | 2.25E-02 |
| 40 | Serine (or cysteine) proteinase inhibitor, clade G, member 1 | Serping1 | NM_009776 | 1.79 | 2.39E-02 |
| 41 | Ubiquitin specific protease 18 | Usp18 | NM_011909.1 | 1.57 | 2.53E-02 |
| 42 | ADP-ribosyltransferase (NAD+; poly (ADP-ribose polymerase)-like 3 | Adprtl3 | NM_145619.2 | 1.53 | 2.58E-02 |
| 43 | Radical S-adenosyl methionine domain containing 2 (2510004L01Rik) | Rsad2 | NM_021384.2 | 1.63 | 2.67E-02 |
| 44 | Cathepsin C | Ctsc | NM_009982.2 | 1.81 | 2.89E-02 |
| 45 | ADP-ribosylation factor-like 11 | Arl11 | NM_177337.3 | 1.55 | 2.93E-02 |
| 46 | RIKEN cDNA 2210408O09 gene (2210408O09Rik) - no info - removed record, BLASTs genomic | 2210408O09Rik | NM_028212.1 | 1.56 | 3.26E-02 |
| 47 | Keratin complex 2, basic, gene 17 | Krt2-17 | NM_010668.1 | 1.97 | 3.69E-02 |
| 48 | Syndecan binding protein (syntenin) 2 | Sdcbp2 | NM_145535.1 | 1.64 | 3.70E-02 |
| 49 | GRP1 (general receptor for phosphoinositides 1)-associated scaffold protein | Grasp | NM_019518.2 | 1.53 | 3.76E-02 |
| 50 | Secretin | Sct | NM_011328.1 | 1.63 | 3.90E-02 |
| 51 | Hypothetical Calcium-binding EGF-like domain containing protein | 6130401L20Rik | AK018073 | -1.58 | 4.52E-02 |
| 52 | Similar to HTPAP protein (LOC381925), , BLASTs genomic | LOC381925 | NM_001080963 | 1.73 | 4.54E-02 |
| 53 | UDP-Gal:betaGlcNAc beta 1,4- galactosyltransferase, polypeptide 1 | B4galt1 | NM_022305.2 | 1.80 | 4.60E-02 |
| 54 | Spermatogenesis associated 13 | Spata13 | XM_901902 | 1.52 | 4.60E-02 |

**Supplementary Table 4.** Symbols and names for the genes included in the IPA network shown in Fig. 1E of the main paper.

Akt, Akt protein; B4GALT1, UDP-Gal:betaGlcNAc, beta 1,4- galactosyltransferase, polypeptide 1;C16 ceramide; CD1D, CD1d molecule; CD68, CD68 molecule; CD74, CD74 molecule major histocompatibility complex, class II invariant chain; CD74- Mhc2a-Mhc2b; CXCL16, chemokine (C-X-C motif) ligand 16; CYP2E1, cytochrome P450, family 2, subfamily E, polypeptide 1; ERK, Erk protein; HLA-DMA, major histocompatibility complex, class II, DMα; HLA-DMB, major histocompatibility complex, class II, DM beta; HLA-DPB1, major histocompatibility complex, class II, DP beta 1; HLA-DQB1, major histocompatibility complex, class II, DQ beta 1; HLADQB2, major histocompatibility complex, class II, DQ beta 2; HLA-DQB3, major histocompatibility complex, class II, DQ beta 3; HLA-DRB1, major histocompatibility complex, class II, DR beta 1; HLA-DRB3, major histocompatibility complex, class II, DR beta 3; HLA-DRB4, major histocompatibility complex, class II, DR beta 4; HLADRB5, major histocompatibility complex, class II, DR beta 5; IFITM1, interferon induced transmembrane protein 1 (9-27); IFITM3, interferon induced transmembrane protein 3 (1-8U); Interferon alpha, Interferon alpha protein; LDL, low density lipoprotein; LOX, lysyl oxidase; MHC Class II; MHC II-&beta; Mhc2 Alpha; NFkB; **NR4A2**, nuclear receptor subfamily 4, group A, member 2; **RBBP4**, retinoblastoma binding protein 4; RSAD2, radical S-adenosyl methionine domain containing 2; SPHK1, sphingosine kinase 1; USP18, ubiquitin specific peptidase 18.

1. **Supplementary Figures**


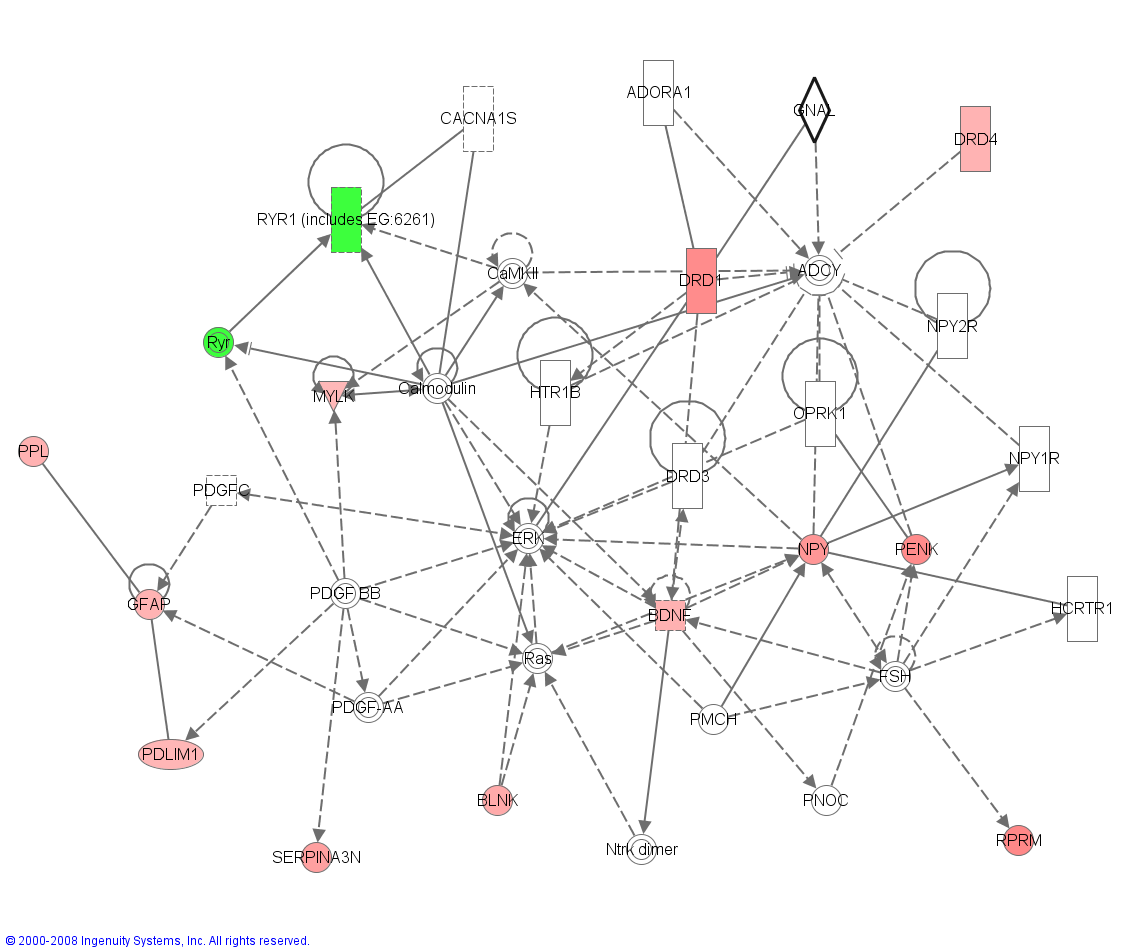
**Supplementary Figure 1**. IPA network 1 including genes differentially expressed in NRG1^tg-type I^ mice at both ages. Explanation of symbols is given after Suppl. Fig. 4.

ADCY, adenylate cyclase; ADORA1, adenosine A1 receptor; BDNF, brain-derived neurotrophic factor; BLNK, B-cell linker; CACNA1S, calcium channel, voltage-dependent, L type, alpha 1S subunit; Calmodulin, Calmodulin protein; CaMKII, calmodulin-dependent protein kinase 2; DRD1, dopamine receptor D1; DRD3, dopamine receptor D3; DRD4, dopamine receptor D4; ERK protein; FSH, follicle stimulating hormone; GFAP, glial fibrillary acidic protein; GNAL, guanine nucleotide binding protein (G protein), alpha activating activity polypeptide; HCRTR1, hypocretin (orexin) receptor 1; HTR1B, 5-hydroxytryptamine (serotonin) receptor 1B; MYLK, myosin light chain kinase; NPY, neuropeptide Y; NPY1R, neuropeptide Y receptor Y1; NPY2R, neuropeptide Y receptor Y2; Ntrk dimer; OPRK1, opioid receptor, kappa 1; PDGF BB, Pdgfb dimer; PDGF-AA, Pdgfa dimer; PDGFC, platelet derived growth factor C; PDLIM1, PDZ and LIM domain 1 (elfin); PENK, proenkephalin; PMCH, pro-melanin-concentrating hormone; PNOC, prepronociceptin; PPL, periplakin; Ras, p21; RPRM, reprimo, TP53 dependent G2 arrest mediator candidate; Ryr, Ryanodine Receptor; RYR1, ryanodine receptor 1 (skeletal), (includes EG:6261); SERPINA3N, serine (or cysteine) peptidase inhibitor, clade A, member 3N.

**Supplementary Figure 2**. IPA network 2 including genes differentially expressed in
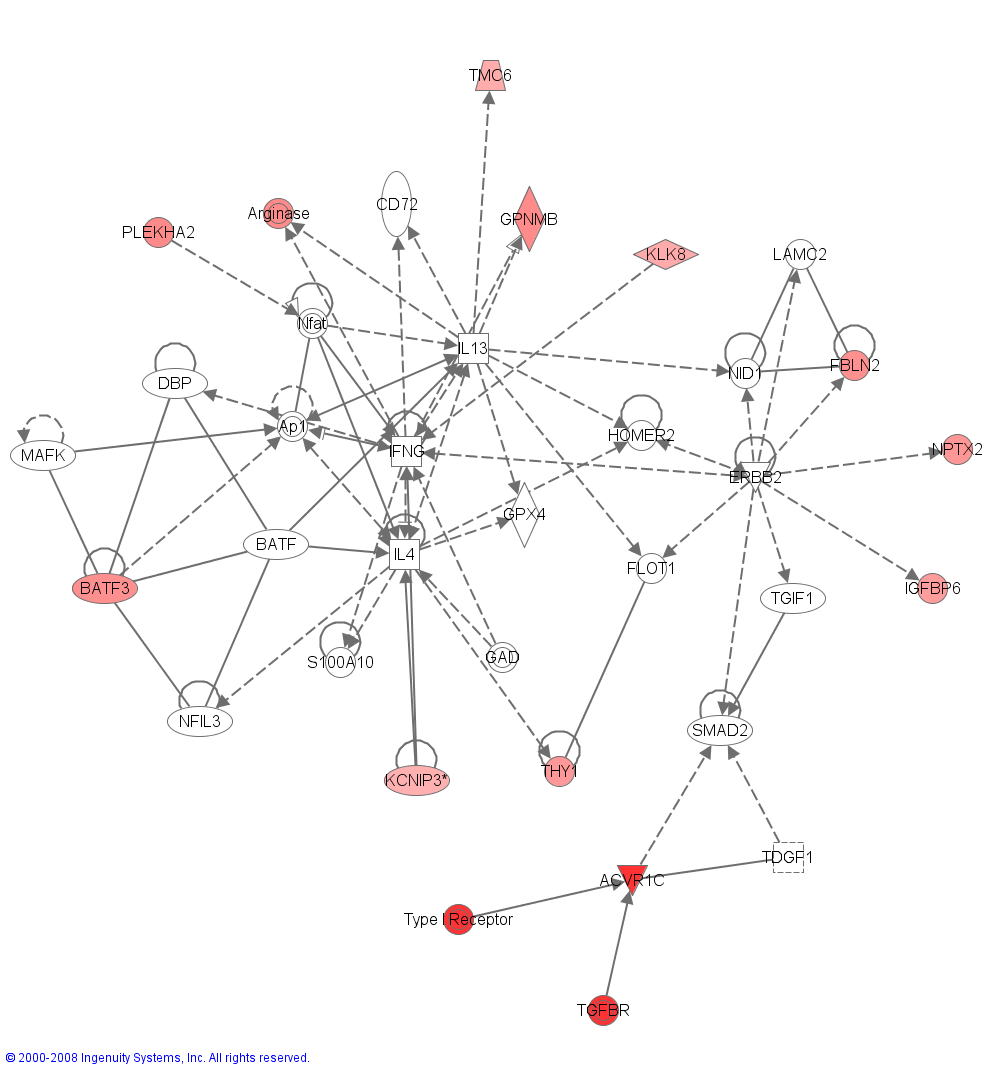
NRG1^tg-type I^ mice at both ages. Explanation of symbols is given after Suppl. Fig. 4.

ACVR1C, activin A receptor, type IC; Ap1, Ap1 protein; Arginase; BATF, basic leucine zipper transcription factor, ATF-like; BATF3, basic leucine zipper transcription factor, ATF-like 3; CD72, CD72 molecule; DBP, D site of albumin promoter (albumin D-box) binding protein; ERBB2, v-erb-b2 erythroblastic leukemia viral oncogene homolog 2, neuro/glioblastoma derived oncogene homolog (avian); FBLN2, fibulin 2; FLOT1, flotillin 1; GAD, L-glutamic acid decarboxylase; GPNMB, glycoprotein (transmembrane) nmb; GPX4, glutathione peroxidase 4 (phospholipid hydroperoxidase), HOMER2, homer homolog 2 (Drosophila); IFNG, interferon, gamma; IGFBP6, insulin-like growth factor binding protein 6; IL13, interleukin 13; IL4, interleukin 4; KCNIP3, Kv channel interacting protein 3, calsenilin; KLK8, kallikrein-related peptidase 8; LAMC, laminin, gamma 2; MAFK, v-maf musculoaponeurotic fibrosarcoma oncogene homolog K (avian); Nfat, Nuclear factor of activated Tcells; NFIL3, nuclear factor, interleukin 3 regulated; NID1, nidogen 1; NPTX2, neuronal pentraxin II; PLEKHA2, pleckstrin homology domain containing, family A (phosphoinositide binding specific) member 2; S100A10, S100 calcium binding protein A10; SMAD2, SMAD family member 2; TDGF1, teratocarcinoma-derived growth factor 1; TGFBR, transforming growth factor beta receptor; TGIF1, TGFB-induced factor homeobox 1; THY1, Thy-1 cell surface antigen; TMC6, transmembrane channel-like 6; Type I Receptor.

**Supplementary Figure 3**. IPA network 1 including genes differentially expressed in young but not old adult NRG1^tg-type I^ mice. Explanation of symbols given after Suppl. Fig. 4.


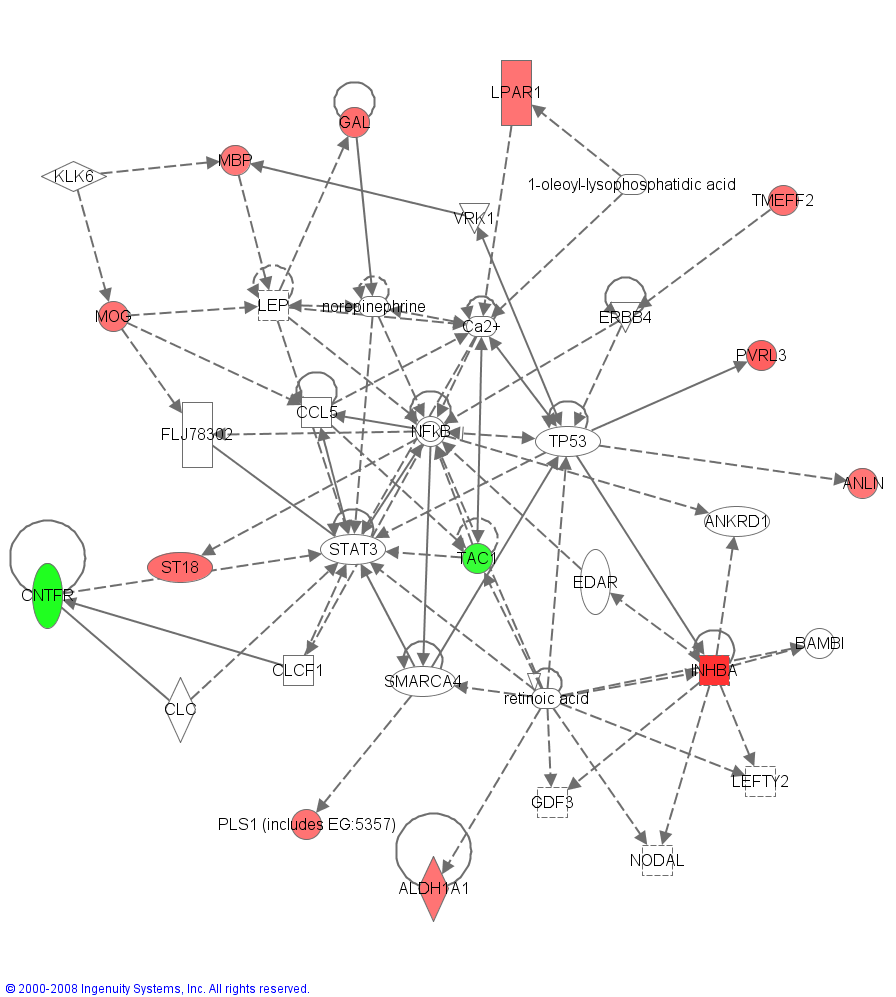
ALDH1A1, aldehyde dehydrogenase 1 family, member A1; ANKRD, ankyrin repeat domain 1 (cardiac muscle); ANLN, anillin, actin binding protein; BAMBI, BMP and activin membrane-bound inhibitor homolog (Xenopus laevis); Ca2+, Calcium ion; CCL5, chemokine (C-C motif) ligand 5; CLC, Charcot-Leyden crystal protein; CLCF1, cardiotrophin-like cytokine factor 1; CNTFR, ciliary neurotrophic factor receptor; EDAR, ectodysplasin A receptor; ERBB4, v-erb-a erythroblastic leukemia viral oncogene homolog 4 (avian); FLJ78302, chemokine (C-C motif) receptor 2-like; GAL, galanin prepropeptide; GDF3, growth differentiation factor 3; INHBA, inhibin, beta A; KLK6, kallikrein-related peptidase 6; LEFTY2, left-right determination factor 2; LEP, leptin; LPAR1, lysophosphatidic acid receptor 1; MBP, myelin basic protein; MOG, myelin oligodendrocyte glycoprotein; NFkB; NODAL, nodal homolog (mouse); norepinephrine; PLS1 (includes EG:5357) plastin 1 (I isoform); PVRL3, poliovirus receptor-related 3; retinoic acid, SMARCA4, SWI/SNF related, matrix associated, actin dependent regulator of chromatin, subfamily a, member 4; ST18, suppression of tumorigenicity 18 (breast carcinoma) (zinc finger protein); STAT3, signal transducer and activator of transcription 3 (acute-phase response factor); TAC1, tachykinin, precursor 1; TMEFF2, transmembrane protein with EGF-like and two follistatin-like domains 2; TP53, tumor protein p53; VRK1, vaccinia related kinase 1.

**Supplementary Figure 4**. IPA network 2 including genes differentially expressed in old but not young adult NRG1^tg-type I^ mice.


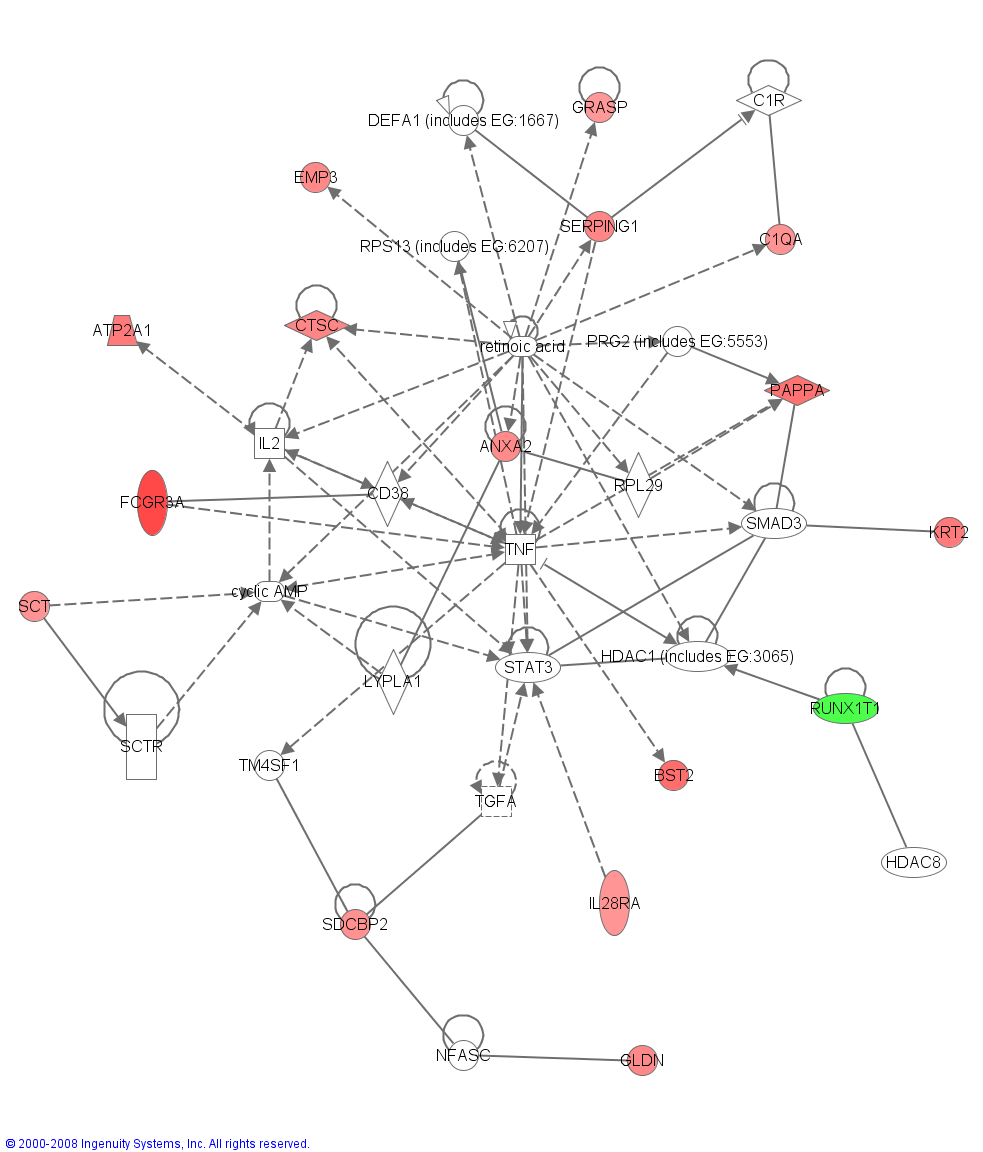


ANXA2, annexin A2; APBA2, amyloid beta (A4) precursor protein-binding, family A, member 2; ATP2A1, ATPase, Ca++ transporting, cardiac muscle, fast twitch 1; BST2, bone marrow stromal cell antigen 2; C1q; C1QA, complement component 1, q subcomponent, A chain; C1R, complement component 1, r subcomponent; CAV1, caveolin 1, caveolae protein, 22kDa; CD38, CD38 molecule; CD68, CD68 molecule; CD99 (includes EG:4267), CD99 molecule; CTSC, cathepsin C; DEFA1 (includes EG:1667), defensin, alpha 1; EMP3, epithelial membrane protein 3; FCGR3A, Fc fragment of IgG, low affinity IIIa, receptor (CD16a); FLNC, filamin C, gamma (actin binding protein 280); GRASP, GRP1 (general receptor for phosphoinositides 1)-associated scaffold protein; IL4, interleukin 4; isopentenyl diphosphate; ITM2B, integral membrane protein 2B; JUN, jun oncogene; KCNH2, potassium voltage-gated channel, subfamily H (eag-related), member 2; MS4A2, membrane-spanning 4-domains, subfamily A, member 2 (Fc fragment of IgE, high affinity I, receptor for; beta polypeptide); Peptidase; PRG2 (includes EG:5553), proteoglycan 2, bone marrow (natural killer cell activator, eosinophil granule major basic protein); PTX3, pentraxin-related gene, rapidly induced by IL-1 beta; retinoic acid; RPL29, ribosomal protein L29; RPS13 (includes EG:6207), ribosomal protein S13; RPS14, ribosomal protein S14; RUNX1T1, runt-related transcription factor 1; translocated to, 1 (cyclin D-related); SCT, secretin; SERPING1, serpin peptidase inhibitor, clade G (C1 inhibitor), member 1, (angioedema, hereditary); TNF, tumor necrosis factor (TNF superfamily, member 2); TNFAIP8, tumor necrosis factor, alpha-induced protein 8.

**Legend for Supplementary Figures 1-4.** The networks comprise nodes (molecules) and their biological relationships by interconnecting lines. Red nodes are more highly expressed in the NRG1^tg-type I^ mice and the green nodes are expressed at a lower level in the NRG1^tg-type I^ mice, with increasing color intensity indicating a greater fold change. White nodes were not included in the uploaded lists but are functionally related to the other differentially expressed genes in the network and added by IPA. Solid lines between nodes indicate a direct interaction between them and dashed lines indicate indirect relationships. A continuous line denotes “binding only”; pointed line, “acts upon” and blunt ended line, “inhibits”. The definitions of the gene symbols are in the legends alongside each figure.

**
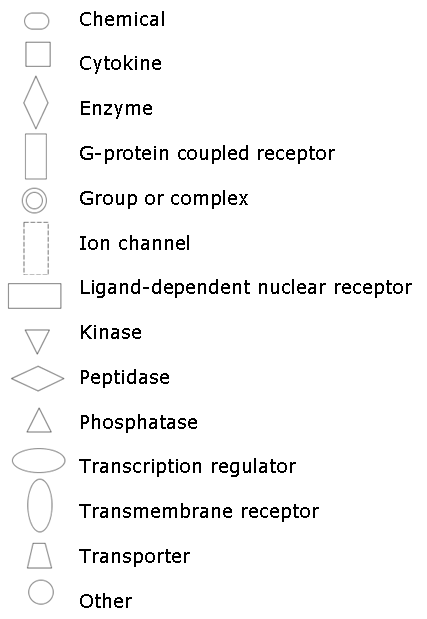
**
